# Non-traumatic fractures following seizures: two case reports

**DOI:** 10.1186/1757-1626-3-30

**Published:** 2010-01-18

**Authors:** Koussay Ach, Ines Slim, Sihem Trimech Ajmi, Molka Chadli Chaieb, Amel Maaroufi Beizig, Larbi Chaieb

**Affiliations:** 1 Department of Endocrinology and Diabetology, Farhat Hached University Hospital, Ibn Jazzar Street, 4002 Soussa, Tunisia

## Abstract

**Introduction:**

Seizures with or without trauma may cause fractures that occur commonly in epileptic seizures. Fracture risk is less reported in non-epileptic seizures. Some metabolic conditions leading to a decrease in bone mineral density may cause fractures secondary to non-epileptic seizure.

**Case presentation:**

We describe two cases of non-traumatic acetabular and vertebrae fractures following seizures without history of epilepsy. They occurred in two male patients, 18 and 48 years old suffering respectively from hypercorticism and poorly controlled diabetes mellitus. Seizures, occurring inside hospital, were secondary to hypertensive encephalopathy crisis with hypokaliemia in the first case and severe hypoglycaemia in the second one. Fracture was promoted by a decrease in mineral bone density caused respectively by hypercorticism and diabetic chronic renal failure.

**Conclusion:**

These observations emphasize that fracture prevention among patients with decreased mineral bone density should include the avoidance of metabolic causes of seizure.

## Introduction

Seizures increase fracture risk [1], occurring in the whole of the skeleton even without trauma [2]. This risk is mainly reported in epileptic seizures because of repeated occurrence of attacks and osteopenia due to the use of anti-epileptic drugs [1]. Literature data have shown that fractures are more related to the seizure itself rather than to the iatrogenic factor [1]. Moreover, some metabolic conditions may lead to a decrease in bone mineral density and hence may be considered as risk factors of fracture when seizures occur. We report here two cases of this unusual situation to resort its eventual prevention.

## Case presentation

### Case report 1

An Arabic 48-year-old man from North Africa (Tunisia) with a ten year history of diabetes mellitus is treated by insulin twice a day for seven years. His diabetes is poorly controlled mainly due to lack of observance. Additionally, he suffered from depression with suicide ideas. Shortly after an overdose of insulin, he developed generalized tonic-clonic seizures that was not followed by trauma and that regressed after parenteral hypertonic glucose serum perfusion.

**Figure 1:**
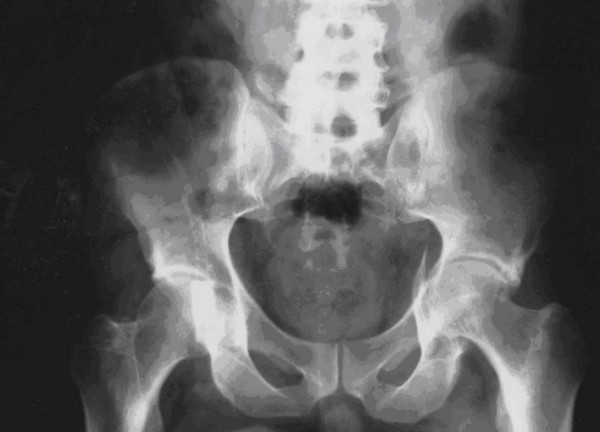
**Radiography of hip after seizures showing left acetabular fracture (Case 1)**.

Laboratory assessment showed a severe hypoglycemia (capillary glycemia at 1.8 mmol/l) and nephrotic syndrome with renal failure (creatininemia at 241 μmol/l). After seizure, the patient presented a disability of the left lower limb. X-ray examination showed a fracture in the left iliac bone localized in the acetabular floor (Figure 1) while brain CT-scan was normal. The patient was referred to orthopedic center where he was treated conservatively. Six months later, the patient was able to walk. Insulin therapy was adjusted aiming to avoid hypoglycemia.

### Case report 2

An Arabic 18-year-old man from North Africa (Tunisia) was referred to our department for suspicion of Cushing syndrome. On physical examination, the patient was obese (BMI at 32 kg/m^2^), he had purplish abdominal striae and hypertension (systolic pressure between 15 and 17 mmHg). Neurological examination was normal.

**Figure 2:**
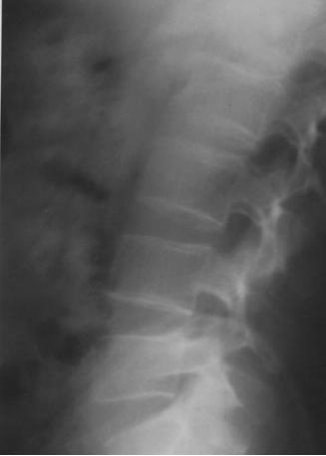
**Radiography of lumbar vertebras before seizures showing bone demineralization (patient n 2)**.

Laboratory examination showed hypokalemia at 2.1 mmol/l confirmed in several samples separately performed while glycemia and calcemia were at normal range. Urinary free cortisol excretion was high (462 μmol/24 h) and was not suppressed after both low and high doses of dexamethasone confirming the diagnosis of Cushing syndrome. X-ray examination (Figure 2) and bone densitometry revealed decrease of bone mineral density with a Z-score of -2. Brain MRI imaging showed a double pituitary adenoma without any other hemispheric lesion. The diagnosis of corticotropic adenoma was retained.

**Figure 3:**
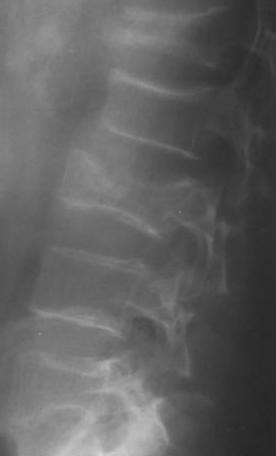
**Radiography of lumbar vertebras after seizures showing fractures (patient n 2)**.

During an acute hypertensive peak at 220 mm Hg, the patient developed tonic-clonic seizures without any trauma. His glycemia was normal (5.6 mmol/l). Seizures regressed after intravenous load of Loxen* and Diazepam* however the patient suffered from mild low back pain. Radiographies identified L_2_, L_3_, L_4 _and L_5 _burst fractures (Figure 3).

Cushing disease was surgically treated with regression of hypertension and weight. Bone mineral density was checked two years later and showed an improved Z-score.

## Discussion

We reported here two cases of hip and vertebrae fractures following non-epileptic generalized tonic-clonic seizures without any trauma or fall. Several data have reported fractures during seizures. Fracture risk is notably increased mainly among epileptic patients. It occurs in 11% of patients mainly within the first two years of diagnosis. This risk is directly related to seizures in 43% of cases in one of the largest series [3].

Fractures concern the whole of skeleton particularly the skull, members, vertebras and the hip and may be multiples, bilateral or associated to other lesions such as dislocations of the shoulder [4,5]. They induce an increase of management cost of epileptic disease [6]. Acetabular fracture observed in the first patient has also been reported and may be serious leading to fatal pelvic haemorrhage [7,8].

Non-epileptic seizures reported in our cases are unusual. In our first case, seizures are related to severe hypoglycaemia, which is common cause of seizures. They were reported in 4.7% of all the hospitalisations for hypoglycaemias especially in old and depressive patients [9]. Concerning the second patient, seizures are caused by encephalopathy hypertensive crisis in addition to hypokaliemia due to hypercorticism. These results are in line with database that emphasizes the role of hypertensive encephalopathy [10], and ionic disorders in seizures such as hypokaliemia and hypocalcaemia [5].

Fractures occurring without any trauma suggest previous bone fragility due to other illness [5]. Bone fragility has been confirmed in our second patient by bone mineral densitometry assessment before seizures.

Our cases concern male young patients who are not at risk of primary osteoporosis. Among classic reported causes of male secondary osteoporosis [11], we can incriminate secondary hyperparathyroidism caused by diabetic renal failure in the first patient [12] and hypercorticism in the second one [13].

Hypercorticism is one of the main diseases that cause osteoporosis leading to fractures. Vertebral bodies and ribs are the typical sites of fracture both in Cushing's syndrome and in patients using glucocorticoid drugs long-term [14]. The frequency of vertebral fractures has ranged from 16 to 20% [14]. Decreased bone mineralization is particularly pronounced in children and in young females with Cushing's syndrome [14]. Interestingly, earlier studies had shown that young and adolescent patients undergoing glucocorticoid therapy may lose bone mass more rapidly than older patients [15]. Fracture risk is higher at the onset of disease and may reveal it. It may also occur during clinical investigations such as in our patient [13] or when diagnosis is delayed [16] leading to definitive bone sequels.

Nevertheless, diabetes mellitus is an unusual cause of osteoporosis and usually we find associated fracture risk factors [17]. Diabetes could play an indirect role through increasing fall risk-related fractures due to feet injuries [18]. Of interest, some prospective data showed that diabetes mellitus is a statistically risk factor of osteoporosis especially in insulin treated diabetes, long history of diabetes or in elderly [19].

The role of metabolic control in osteopenia is still not well established despite that some data suggest that insulin, growth factors or osmotic factors such as extra-cellular glucose levels may play a significant role in the osteoblast metabolism [20].

## Conclusion

These cases demonstrate the possibility of non-traumatic fracture following seizure. Diabetes mellitus and hypercorticism may cause, even in males, osteoporosis and subsequently are considered as risk factors of seizures and fractures. Importantly, these conditions may be well-managed to prevent osteoporosis. We insist not only on avoiding falls and trauma but also on preventing metabolic causes of seizures such as hypoglycaemia, ionic disturbances and hypertensive encephalopathy.

## Consent

Written informed consent was obtained from the patient for publication of this case report and accompanying images. A copy of the written consent is available for review from the journal's Editor-in-Chief.

## Competing interests

The authors declare that they have no competing interests.

## Authors' contributions

KA, IS, STA followed the patients reported here. KA, IS wrote the paper, MCC, AMB, LC reviewed the paper.

## References

[B1] Vestergaard P Tigaran S Rejnmark L Tigaran C Dam M Mosekilde L Fracture risk is increased in epilepsy Acta Neurol Scand 1999 99 269 275 10.1111/j.1600-0404.1999.tb00675.x 10348155

[B2] Takahashi T Tominaga T Shamoto H Shimizu H Yoshimoto T Seizure-induced thoracic spine compression fracture: case report Surg Neurol 2002 58 214 216 10.1016/S0090-3019(02)00837-6 12480222

[B3] Persson HB Alberts KA Farahmand BY Tomson T Risk of extremity fractures in adult outpatients with epilepsy Epilepsia 2002 43 768 772 10.1046/j.1528-1157.2002.15801.x 12102682

[B4] Heckmann JG Stangl R Erbguth F Rutherford H Neundorfer B Non traumatic seizure-associated bilateral fractures of the head of the humerus Intensive Care Med 1999 25 548 549 10.1007/PL00003777 10401960

[B5] Gosens T Poels PJ Rondhuis JJ Posterior dislocation fractures of the shoulder in seizure disorders - two case reports and a review of literature Seizure 2000 9 446 448 10.1053/seiz.2000.0418 10986005

[B6] Davidson DL Mac Donald S The costs of trauma caused by seizures: can they be reduced? Seizure 2002 11 344 347 10.1053/seiz.2001.0620 12076109

[B7] Sikkink CJ Tol A van der Unilateral transverse acetabular fracture with medial displacement of the femoral head after an epileptic seizure J Trauma 2000 48 777 778 10.1097/00005373-200004000-00032 10780618

[B8] Hughes CA O'Briain D Sudden death from pelvic haemorrhage after bilateral central fracture dislocations of the hip due to an epileptic seizure Am J Forensic Med Pathol 2000 21 380 384 10.1097/00000433-200012000-00017 11111802

[B9] Stepka M Rogala H Czyzyk A Hypoglycaemia: a major problem in the management of diabetes in the elderly Aging (Milano) 1993 5 117 121 8323998 10.1007/BF03324137

[B10] Zgurzynski P Manno M Coccygeal fracture, constipation, convulsion, and confusion: a case report of malignant hypertension in a child Pediatr Emerg Care 1999 15 425 428 10.1097/00006565-199912000-00016 10608334

[B11] Seeman E Osteoporosis in men Baillieres Clin Rheumatol 1997 11 613 629 10.1016/S0950-3579(97)80023-4 9367040

[B12] Akcali O Kosay C Gunal I Alici E Bilateral trochanteric fractures of the femur in a patient with chronic renal failure Int Orthop 2000 24 179 180 10.1007/s002640000146 10990395 PMC3619882

[B13] Mancini T Doga M Mazziotti G Giustina A Cushing's syndrome and bone Pituitary 2004 7 243 246 10.1007/s11102-005-1051-2 16010458

[B14] Khanine V Fournier JJ Requeda E Luton JP Simon F Crouzet J Osteoporotic fractures at presentation of Cushing's disease: two case reports and a literature review Joint Bone Spine 2000 67 341 345 10963086

[B15] Ruegsegger P Medici T Anlider M Corticosteroid-induced bone loss: a longituidal study of alternate day therapy in patients with bronchial asthma using quantitative computed tomography Eur J Clin Pharmacol 1983 25 615 620 10.1007/BF00542348 6662161

[B16] Yoshihara A Okubo Y Tanabe A Sata A Nishimaki M Kawamata T Kubo O Hori T Takano K A Juvenile Case of Cushing's Disease Incidentally Discovered with Multiple Bone Fractures Intern Med 2007 46 583 587 10.2169/internalmedicine.46.1824 17473494

[B17] Forsen L Meyer HE Midthjell K Edna TH Diabetes mellitus and the incidence of hip fracture: results from the Nord-Trondelag Health Survey Diabetologia 1999 42 920 925 10.1007/s001250051248 10491750

[B18] Wallace C Reiber GE LeMaster J Smith DG Sullivan K Hayes S Vath C Incidence of falls, risk factors for falls, and fall-related fractures in individuals with diabetes and a prior foot ulcer Diabetes Care 2002 25 1983 1986 10.2337/diacare.25.11.1983 12401743

[B19] Nicodemus KK Folsom AR Iowa Women's Health Study. Type 1 and type 2 diabetes and incident hip fractures in postmenopausal women Diabetes Care 2001 24 1192 1197 10.2337/diacare.24.7.1192 11423501

[B20] Leidig-Bruckner G Ziegler R Diabetes mellitus a risk for osteoporosis? Exp Clin Endocrinol Diabetes 2001 109 Suppl 2 S493 S514 10.1055/s-2001-18605 11460594

